# Maternal DHA Supplementation during Pregnancy and Lactation in the Rat Protects the Offspring against High-Calorie Diet-Induced Hepatic Steatosis

**DOI:** 10.3390/nu13093075

**Published:** 2021-08-31

**Authors:** Amran Daher-Abdi, Sandra Olvera Hernández, Luis Antonio Reyes Castro, Carla Elena Mezo-González, Mikaël Croyal, Juan Antonio García-Santillán, Khadija Ouguerram, Elena Zambrano, Francisco Bolaños-Jiménez

**Affiliations:** 1 UMR Physiologie des Adaptations Nutritionnelles, INRAE—Université de Nantes, 44096 Nantes, France; amran.daherabdi@gmail.com (A.D.-A.); olvera.sandra@uabc.edu.mx (S.O.H.); lafe_mat@hotmail.com (L.A.R.C.); carla.mezo-gonzalez@etu.univ-nantes.fr (C.E.M.-G.); juantgarsan@hotmail.com (J.A.G.-S.); khadija.ouguerram@univ-nantes.fr (K.O.); 2 Medical and Psychology School, Autonomous University of Baja California, Tijuana 21100, Mexico; 3 Reproductive Biology Department, Instituto Nacional de Ciencias Médicas y Nutrición Salvador Zubirán, Mexico City 14080, Mexico; zamgon@yahoo.com.mx; 4 CRNH-O Mass Spectrometry Core Facility, 8 Quai Moncousu, 44000 Nantes, France; Mikael.Croyal@univ-nantes.fr; 5 Université de Nantes, CNRS, INSERM, L’institut du Thorax, 8 Quai Moncousu, 44000 Nantes, France; 6 Université de Nantes, CHU Nantes, Inserm, CNRS, SFR Santé, Inserm UMS 016, CNRS UMS 3556, 8 Quai Moncousu, 44000 Nantes, France

**Keywords:** developmental programming, DHA, obesity, hepatic steatosis

## Abstract

Maternal supplementation during pregnancy with docosahexaenoic acid (DHA) is internationally recommended to avoid postpartum maternal depression in the mother and improve cognitive and neurological outcomes in the offspring. This study was aimed at determining whether this nutritional intervention, in the rat, protects the offspring against the development of obesity and its associated metabolic disorders. Pregnant Wistar rats received an extract of fish oil enriched in DHA or saline (SAL) as placebo by mouth from the beginning of gestation to the end of lactation. At weaning, pups were fed standard chow or a free-choice, high-fat, high-sugar (fc-HFHS) diet. Compared to animals fed standard chow, rats exposed to the fc-HFHS diet exhibited increased body weight, liver weight, body fat and leptin in serum independently of saline or DHA maternal supplementation. Nevertheless, maternal DHA supplementation prevented both the glucose intolerance and the rise in serum insulin resulting from consumption of the fc-HFHS diet. In addition, animals from the DHA-fc-HFHS diet group showed decreased hepatic triglyceride accumulation compared to SAL-fc-HFHS rats. The beneficial effects on glucose homeostasis declined with age in male rats. Yet, the preventive action against hepatic steatosis was still present in 6-month-old animals of both sexes and was associated with decreased hepatic expression of lipogenic genes. The results of the present work show that maternal DHA supplementation during pregnancy programs a healthy phenotype into the offspring that was protective against the deleterious effects of an obesogenic diet.

## 1. Introduction

It has well established that the nutritional status of the mother during gestation and lactation is a key determinant factor in the long-term health of the offspring. Epidemiological observations in humans show that children of obese mothers or those born to mothers who suffered from nutritional deficiencies during pregnancy are at greater risk of developing cognitive deficits and metabolic disorders than children of adequately nourished pregnant mothers [1,2,3,4]. These epidemiological observations have been corroborated by the results of numerous experimental studies in various animal species and conceptualized under the hypothesis of the Developmental Origins of Health and Disease (DOHaD), also known as metabolic programming or nutritional programming [5,6,7]. According to this hypothesis, an imbalanced nutritional environment during in utero development and/or neonatal life sensitizes offspring to developing metabolic and mental diseases via epigenetic mechanisms that translate the effects of early nutritional events, leading to long-term changes in behavior and energy homeostasis. However, just as an unhealthy maternal diet can lead to adverse health consequences in the offspring, maternal supplementation with specific nutrients during pregnancy can potentially reverse and/or protect against the detrimental effects of metabolic programming [8,9]. In this respect, maternal supplementation with docosahexaenoic acid (DHA) is a common clinical practice.

DHA is a polyunsaturated fatty acid that is found in large quantities in fish oil and seaweed. It is essential for the optimal development of the brain. However, it is only produced in marginal quantities by the body—hence, the recommendation to supplement the diet of pregnant women with DHA [10]. This nutritional intervention is supposed to reduce the risk of cerebral palsy, avoid premature birth and postpartum maternal depression, and increase birth weight [10,11,12,13]. Maternal DHA supplementation, however, has been extended beyond its use in preventing developmental defects. Consumption of this fatty acid is also believed to improve the cognitive development of the child. Substantial evidence indicates that children of mothers who received DHA during pregnancy exhibit better learning and cognitive indices than children of placebo-supplemented mothers [14,15]. Nevertheless, no benefits on offspring’s neurodevelopment of maternal DHA supplementation have also been documented [16].

Clinical studies have also shown that supplementing the diet with DHA and/or eicosapentaenoic acid (EPA) reduces body fat [17,18], plasma triglycerides [19] and fatty acid accretion in liver [20,21]. These effects would be mediated by increased hepatic fatty oxidation and plasma lipoprotein lipase activity associated with reduced hepatic lipogenesis and very-low-density lipoprotein (VLDL) production [22,23,24,25]. Recently, it was proposed that the production of N-acyl taurines could also be involved [26]. Lower body fat accretion and reduced dyslipidemia have also been observed in obese mice and rats fed a high-fat diet supplemented with DHA/EPA [27,28]. On the basis of these findings, we hypothesized that maternal DHA supplementation could not only improve a child’s cognitive development but also promote a healthy metabolic state that would exert a protective effect against the deleterious effects of an obesogenic diet. Therefore, this study aimed to evaluate (in rat offspring) the potential protective effects of maternal DHA supplementation during pregnancy and lactation against the metabolic alterations induced by a high-calorie diet and to uncover the involved molecular mechanisms.

## 2. Materials and Methods

### 2.1. Animals and Nutritional Interventions

**Figure 1:**
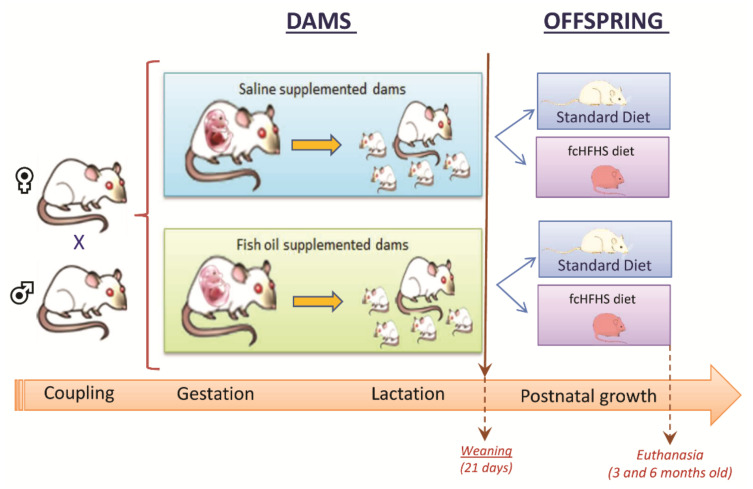
General scheme of the nutritional intervention.

The workflow of the study is illustrated in Figure 1. Founder (F0) female and male Wistar rats of three months of age were obtained from Janvier Labs (Le Genest Saint Isle, France) and maintained under an inversed 12 h/12 h dark/light cycle (lights off at 7:00 AM) at 22 ± 1 °C with food and water ad libitum. After an adaptation period of ten days, females in natural proestrus were allowed to copulate with a male until at least two ejaculations were displayed. From the day after mating until the end of lactation, pregnant rats were housed individually and supplemented by mouth every day with an extract of fish oil containing 77 mg/g of EPA and 521 mg/g of DHA in the form of triglycerides (Omegavie^®^ DHA 60 TG Qualitysilver^®^, Polaris, Quimper, France). The DHA formulation was contained within capsules (intended for human use) made of fish gelatin, glycerol and water which were protected from heat and light. The extract of fish oil was withdrawn by puncturing the capsule with a 1 mL syringe just before oral administration. DHA and EPA were thus protected from oxidation throughout storage and administration. The purity of the formulation was confirmed by mass spectrometric quantification of the concentration of DHA and EPA as described in Section 2.4. The amount of delivered oil was adjusted to provide pregnant rats a daily dose of 175 mg of DHA per Kg. This corresponded, in average, to 0.85% of their daily caloric intake during the gestation period and to 0.41% of their daily caloric intake during lactation. One day after delivery, litter size was adjusted to eight pups per litter maintaining a 1:1 male to female ratio. At weaning (21 days), pups were divided into two groups to be fed either standard chow (A04, SAFE, Augy, France, controls) or a free-choice high-fat high-sugar diet (fc-HFHS) in addition to standard chow and tap water (obese), as described by La Fleur et al. [29], with minor modifications. The fc-HFHS diet consisted of powdered standard chow mixed with pig fat in the proportion of 30 g of fat per 70 g of food and a 30% sugar solution prepared with sweetened condensed milk. Four experimental groups were thus constituted: offspring of saline-supplemented dams fed standard chow after weaning (SAL-STD); offspring of saline-supplemented dams exposed to fcHFHS diet (SAL-fcHFHS diet); offspring of DHA-supplemented dams fed standard chow (DHA-STD); offspring of DHA-supplemented dams exposed to the fcHFHS diet (DHA-fcHFHS diet). The detailed composition of the standard chow can be found on the website: https://safe-lab.com/safe-wAssets/docs/product-data-sheets/diets/safe_a04_ds.pdf (accessed 29 August 2021). Its energy value was 3.15 kcal/g, while that of the standard chow mixed with pork fat was 5.10 kcal/g. The caloric value of the sweetened water solution was 1.75 kcal/mL. A total of 16 dams (8 supplemented with saline and 8 supplemented with DHA) were used. Two pups were selected at random from each litter to yield a final number of 16 saline- and 16 DHA-supplemented pups. Care was taken to split the two pups of the same dam into different experimental groups (fed standard chow or exposed to fcHFHS diet), such that the presented results corresponded to the combined data from eight animals born to eight different dams. Pups were housed four per cage until they reached 300 g of body weight and two per cage thereafter.

### 2.2. Measurement of Body Weight and Food Intake

The weight of each dam was recorded every day throughout gestation and lactation to adjust the amount of DHA administered in relation to their body weight. Likewise, to determine the caloric contribution of DHA administration to daily energy consumption, the daily food consumption of the dams was determined by measuring the difference between the amount of pellet-food provided at the onset of their dark cycle and the amount of food remaining 24 h later. Care was taken to check through the sawdust in each cage to collect any spilled pellet of food.

### 2.3. Collection of Biological Samples and Euthanasia

Blood samples were collected by lateral tail vein puncture from dams on day 20 of gestation and at weaning, from supernumerary pups euthanized at the time of litter size adjustment, and from offspring at weaning and euthanasia. Sampling of the mothers took place before the oral administration of DHA. In a first series of experiments, male animals of 3 months of age were used. Thereafter, the same types of analyses were carried out in 6-month-old male and female rats. In both cases, animals were sacrificed by cervical dislocation under isoflurane anesthesia at the beginning of the dark phase of their light/dark cycle. The liver was carefully dissected, immediately frozen in liquid nitrogen and kept at −80 °C until analysis. Mediastinal and abdominal (omental, perirenal, retroperitoneal, epididymal, periovarian, perivesical, and parametrial) fat depots were dissected, weighed and summed to provide a measure of body fat.

### 2.4. Quantification of DHA and EPA

DHA and EPA concentrations in serum and in Omegavie^®^ Qualitysilver capsules were determined by liquid chromatography-tandem mass spectrometry (LC-MS/MS). All solvents used were LC-MS grade and were purchased from Biosolve (Valkenswaard, Netherlands). Standard compounds were obtained from Sigma Aldrich (Saint-Quentin Fallavier, France). A pool of reference standard solutions was prepared and serially diluted in ethanol to obtain seven standard solutions ranging from 0.05 to 80 µmol/L. Exogenous internal standard (2H5-DHA, 50 µL diluted at 20 µmol/L in ethanol) was added to 100 µL of standard solutions and serum samples. Then, 850 µL of a methanol/chloroform mixture (2:1; *v*:*v*) were added and samples were centrifuged for 10 min at 10,000× *g* (4 °C). Supernatants were transferred to vials and dried under a gentle stream of nitrogen. Dried samples were finally resuspended with 100 µL of an isopropanol/acetonitrile/water mixture (30:65:5; *v*:*v*:*v*). LC-MS/MS analyses were performed on a Xevo^®^ TQD mass spectrometer with an electrospray interface and an Acquity H-Class^®^ UPLCTM device (Waters Corporation, Milford, MA, USA). Samples (5 µL) were injected onto a CSH-C18 column (1.7 µm, 2.1 × 100 mm, Waters Corporation) held at 60 °C. Compounds were separated using a linear gradient of mobile phase B (90:10; *v*:*v*; isopropanol:acetonitrile containing 0.1% formic acid and 10 mmol/L ammonium acetate) in mobile phase A (60:40; *v*:*v*; acetonitrile:water; 10 mmol/L ammonium acetate, 0.1% formic acid) at a flow rate of 400 µL/min. Mobile phase B was linearly increased from 40% to 95% for 8 min, kept constant for 1 min, returned to the initial condition over 1 min, and then kept constant for 1.5 min before the next injection. Targeted compounds were detected by the mass spectrometer with the electrospray interface operating in the negative ion mode capillary voltage, 2.5 kV; desolvatation gas (N2) flow and temperature, 1000 L/h and 450 °C; source temperature, 150 °C. The multiple reaction monitoring mode was applied for MS/MS detection with the following m/z transitions: EPA, 301.2 → 257.2; DHA, 327.2 → 283.4; and 2H5-DHA, 332.2 →288.3. Cone and collision energy were set at 35 V and 15 eV, respectively. Chromatographic peak area ratios between EPA or DHA and 2H5-DHA constituted the detector responses. Standard solutions were used to plot calibration curves for quantification. The linearity was expressed by the mean R^2^ which was greater than 0.999 for both compounds (linear regression, 1/× weighting, origin excluded).

### 2.5. Serum Metabolite Determinations

Animals were submitted one week before sacrifice to an oral glucose tolerance test after a fast of 8 h initiated at the beginning of the dark phase of their light/dark cycle. A drop of blood was taken immediately before administration of glucose and 20, 40, 80, and 120 min after by severing the tip of the tail. Blood glucose was determined with a blood glucose monitor (Accu-Check^®^ Active, Roche Diagnostics, Vercors, Meylan, France). To determine the glucose disappearance rate (kG), the area under the curve (AUC) was calculated using the value before the administration of glucose as base line. Serum from blood collected at the time of sacrifice was assayed for insulin and leptin using assay kits from Millipore (Billerica, MA, USA). Enzymatic diagnostic kits from BioMérieux (Craponne, France), were used to quantify the serum levels of triglycerides, glucose and cholesterol.

### 2.6. Quantification of Liver Triglycerides and Cholesterol

200 mg of liver were homogenized in 4 mL of a chloroform/methanol (2:1) solution. After centrifugation of the extract at 4 °C and 2500× *g* during 15 min, the supernatant was collected and evaporated to dryness. The residue was subsequently reconstituted in 300 µL of a solution of isopropyl alcohol containing 10% Triton X and centrifuged at 5000× *g* for 15 min. The resulting supernatant was used for the determination of triglycerides and cholesterol content using enzymatic diagnostic kits from BioMérieux (Craponne, France).

### 2.7. Real-Time Quantitative RT-PCR Experiments

Total RNA was extracted using Trizol reagent (Invitrogen) and treated with DNAse/RNAse-free for 30 min at 37 °C. Afterwards, 1.5 μg of purified RNA was reversed transcribed using Superscript II RNAseH-Reverse-Transcriptase (Invitrogen) in a total volume of 20 μL, and the resulting cDNA was diluted 40-fold in DNAse and RNAse free water. Thereafter, 5 μL of each cDNA diluted sample was used as template for PCR amplification with 18S or β2-microglobulin as housekeeping genes and SYBR Green (Biorad, France) as fluorogenic intercalating dye. PCR was performed with the iCycler iQ detection system (BioRad Laboratories, Hercules, CA, USA), using the following parameters: an initial denaturation step of 5 min at 95 °C, followed by 45 cycles of 30 s at 95 °C, and 30 s at 60 °C. Primers used for the amplifications are presented in Table 1.

### 2.8. Data Analysis

The relative expression levels of the mRNAs in the different samples were calculated using the 2ΔΔCT method. GraphPad Prism 9 Software (GraphPad, Inc., San Diego, CA, USA) was used to perform all statistical analyses. Data were first checked for normality using the Shapiro–Wilk normality test. Statistical differences between obese and control offspring born to saline-supplemented dams were evaluated by unpaired Student’s *t*-test. The differences in the effects of maternal DHA supplementation, or between males and females, were determined by two-way ANOVA followed by Sidak’s multiple comparison test using maternal supplementation (saline or DHA), postnatal diet (chow or FcHFHS diet), or sex as factors. Experimental results were expressed as mean ± S.E.M. Statistical significance was set at *p* < 0.05.

## 3. Results

### 3.1. DHA Supplementation Does Not Affect Maternal Body Weight Gain or Food Intake during Pregnancy nor the Offspring’s Growth Profile

**Figure 2:**
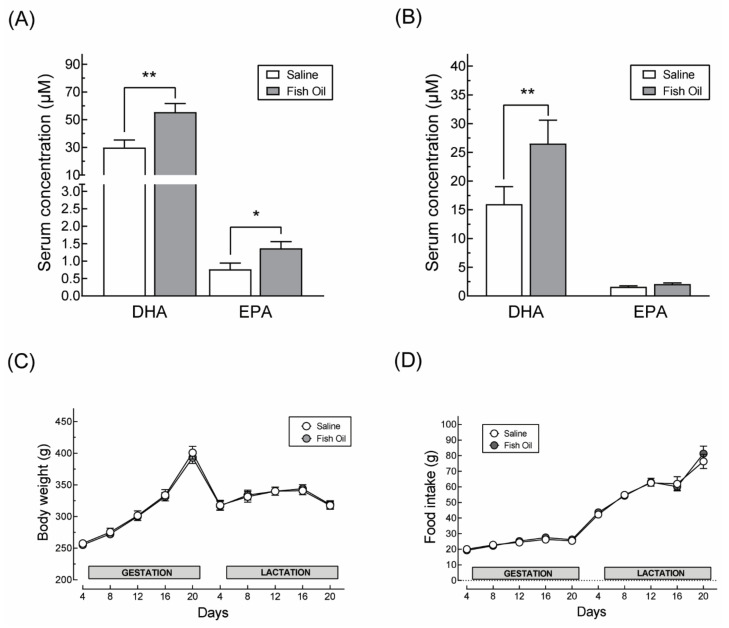
Effects of fish oil extract supplementation on maternal serum DHA and EPA at gestation day 20 (**A**) and at weaning (**B**). Note that the increased circulating levels of DHA and EPA has no impact on the body weight (**C**) and food consumption pattern (**D**) of the dams. * *p* < 0.05; ** *p* < 0.01 as determined by Student’s *t*-test.

Supplementation with fish oil extract increased the maternal blood levels of DHA in relation to saline-supplemented dams from 30 µM to 55 µM at the end of the gestation period and from 16 µM to 26 µM at weaning (Figure 2A,B). Serum EPA levels increased only during the gestation period, from 0.71 µM to 1.56 µM (Figure 2A). DHA-supplemented dams showed the same pattern of weight gain and food intake during gestation and lactation as their saline-supplemented counterparts (Figure 2C,D).

**Figure 3:**
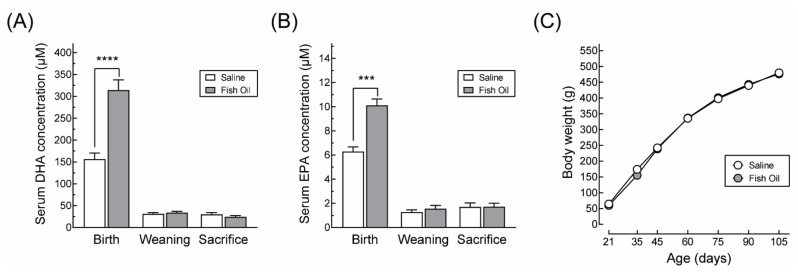
DHA (**A**) and EPA (**B**) levels in serum from offspring born to dams supplemented during gestation and lactation with saline or with a fish oil extract. At birth, offspring from supplemented dams exhibited increased circulating levels of DHA and EPA but showed no differences in body weight gain during lactation (**C**). *** *p* < 0.001; **** *p* < 0.0001 (Student’s *t*-test).

It is well established that the concentrations of fatty acids, including DHA, increase during pregnancy independently of the diet and decrease postpartum in both humans [30,31] and rats [32,33]. Moreover, although the levels of DHA in the offspring after birth are determined by the DHA supplied by the mother via the breast milk, this provision ceases between the 16th and 17th day of life when the offspring start to ingest solid food and, as a result, their diet becomes independent of that of their mother. In agreement with these observations, the offspring of mothers supplemented with fish oil extract showed an increase in the serum concentration of DHA and EPA only at birth (Figure 3A,B). Otherwise, their growth profile was identical to that of the animals from saline-supplemented dams (Figure 3C).

### 3.2. Impact of Maternal DHA Supplementation on Male Offspring at Three Months

#### 3.2.1. Anthropometric Characteristics and Serum Metabolite Profile

**Figure 4:**
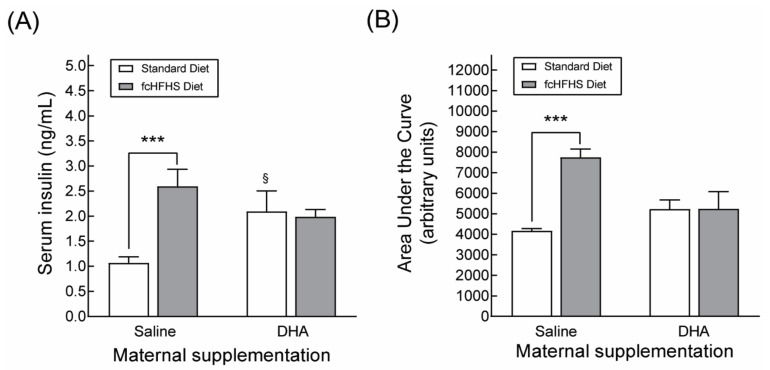
DHA maternal supplementation during gestation and lactation protects the offspring against insulinemia (**A**) and glucose intolerance (**B**) induced by the consumption of a high-calorie diet. Data correspond to the serum levels of insulin under ad libitum feeding conditions. The areas under the curve derive from an oral glucose tolerance test performed in 3-month-old male rats after a fasting period of 8 h. *** *p* < 0.001 (Student’s *t*-test); ^§^ *p* < 0.05 compared to rats fed standard chow born to saline-supplemented dams as determined by two-way ANOVA using maternal supplementation and postnatal diet as factors.

To assess the potential protective effects of maternal DHA supplementation against obesity-induced metabolic alterations in the offspring, the progeny from saline- or DHA-supplemented dams were subdivided into two groups at weaning to be fed standard chow or to be exposed to a diet allowing them to freely choose between standard chow or food high in sugar and fat (FcHFHS Diet). This experimental protocol mimics the conditions of free choice feeding in humans and results in hyperglycemia, hyperinsulinemia and glucose intolerance [31]. 3-month-old male rats exposed to the FcHFHS diet gained more body weight and accumulated more body fat than rats fed standard chow, regardless of whether their mothers were supplemented with saline or DHA (Table 2). Similarly, animals born to dams supplemented with DHA or with saline who were exposed to the FcHSHF diet exhibited elevated circulating levels of leptin and triglycerides compared to animals fed standard chow (Table 2). These results indicated that maternal DHA supplementation had no protective effects on the increased body weight, fat accretion and hyperlipidemia associated with obesity. In contrast, circulating insulin levels and glucose intolerance were reduced in obese animals born to DHA-supplemented dams in comparison to the SAL-fcHFHS diet group (Figure 4A,B). Otherwise, no differences in blood glucose and cholesterol levels were observed between control and obese animals.

#### 3.2.2. Hepatic Lipid Accumulation

**Figure 5:**
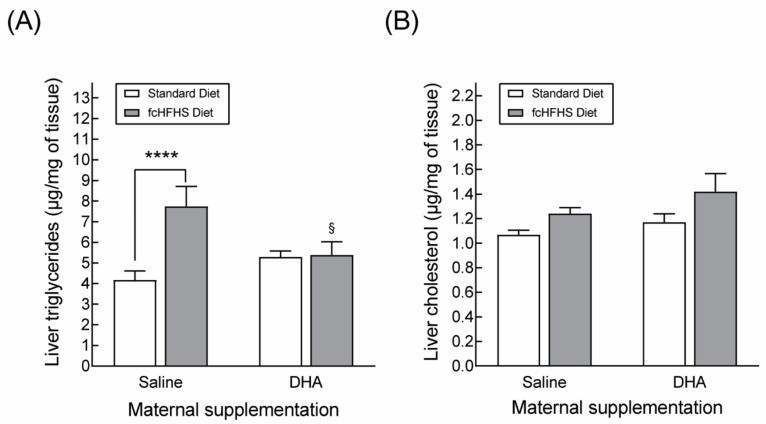
Protective effects of maternal DHA supplementation against obesity-induced hepatic steatosis in the offspring. Data correspond to the concentrations of triglycerides (**A**) and cholesterol (**B**) determined in liver samples from 3-month-old male rats born to saline- or DHA-supplemented dams. The offspring were fed standard chow or rendered obese by exposure to a fcHFHS diet from weaning. **** *p* < 0.0001 (Student’s *t*-test); ^§^ *p* < 0.05 compared to rats born to saline-supplemented dams exposed to the fcHFHS diet after weaning (two-way ANOVA using maternal supplementation and postnatal diet as factors).

It has been shown that DHA supplementation in parallel with high-calorie feeding protects against the development of obesity-associated liver steatosis [34,35]. Therefore, in order to further investigate the beneficial effects of maternal DHA supplementation, we assessed the accumulation of triglycerides and cholesterol in liver samples from our four experimental groups. Rats on the FcHFHS diet born to saline-supplemented dams displayed a higher concentration of triglycerides in the liver compared to the SAL-STD group, but there were no differences in hepatic triglycerides’ concentration between animals of the DHA-STD and DHA-fcHFHS diet groups (Figure 5A). In addition, obese animals born to saline-supplemented dams showed increased concentrations of cholesterol in the liver compared to rats of the SAL-STD diet group (Figure 5B). However, no significant differences in hepatic cholesterol were observed between obese and non-obese animals born to DHA-supplemented dams.

#### 3.2.3. Hepatic Gene Expression

**Figure 6:**
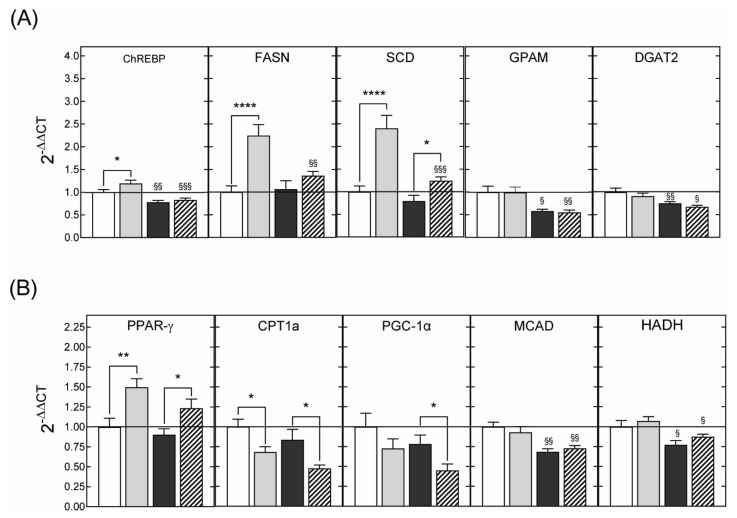
Expression levels of mRNAs encoding proteins involved in de novo lipogenesis (**A**) or fatty acid oxidation (**B**) in liver samples from 3-month-old male rats born to dams supplemented with saline or with DHA during gestation and lactation. The offspring were fed standard chow or rendered obese by exposure to a fcHFHS diet from weaning. Variations in gene expression were calculated by the 2^-ΔΔCT^ method using the expression in animals of the SAL-STD group (white bars) as a calibrator. Grey bars, SAL-fcHFHS group; black bars, DHA-STD group; stripped bars, DHA-fcHFHS diet group. * *p* < 0.05; ** *p* < 0.01; **** *p* < 0.0001 (Student’s *t*-test); ^§^ *p*< 0.05 ^§§^ *p* < 0.01; ^§§§^ *p* < 0.001 compared to animals born to saline supplemented dams that were exposed to the same diet (standard or fcHFHS diet), after weaning (two-way ANOVA with maternal supplementation and postnatal diet as factors).

In order to determine the mechanisms underpinning the beneficial effects of DHA supplementation, the hepatic expression levels of genes involved in glucose and lipid metabolism were examined. The results of these analyses showed that the protective effects of maternal DHA supplementation against hepatic steatosis were associated with decreased expression of mRNAs encoding for genes that positively regulate de novo lipogenesis and triglyceride synthesis (Figure 6A). Similarly, hepatic expression of genes promoting β-oxidation was downregulated by maternal DHA supplementation (Figure 6B).

### 3.3. Impact of Maternal DHA Supplementation on the Offspring at Six Months

The deleterious effects of metabolic programming often worsen with age and might be sex-dependent. With the aim of determining the persistence of the protective effects of DHA and potential sex differences in response to maternal DHA supplementation, a new series of experiments was conducted in which 6-month-old male and female animals born to saline- or DHA-supplemented dams were examined.

#### 3.3.1. Anthropometric Characteristics and Serum Metabolite Profile

As expected, 6-month-old male animals exposed to the fcHFHS diet since weaning, showed an aggravation of their metabolic state compared to 3-month-old male animals exposed to the same diet. This was reflected in greater increases in body weight (+24%), body fat (+9%) and liver triglycerides (+316%) and cholesterol (+299%) in response to the fcHFHS diet (Table 3). Female rats exposed to the fcHFHS diet also showed significant metabolic alterations, some of which exceeded those observed in obese male rats in severity. This was particularly notable with respect to serum triglyceride levels (Table 3).

**Figure 7:**
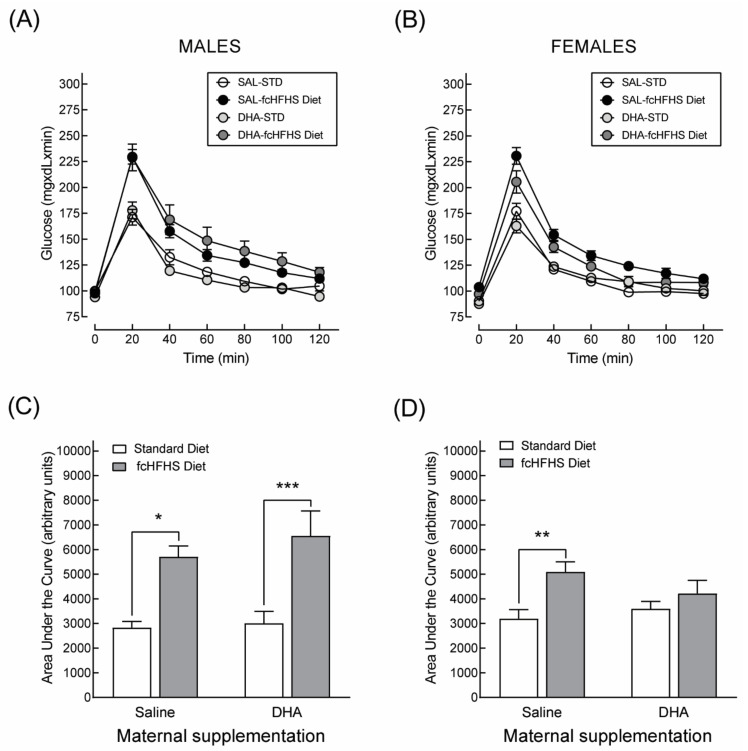
Long-term protective effects of maternal supplementation with DHA during gestation and lactation against high-calorie diet induced glucose intolerance. Data correspond to the curves (**A**,**B**) and the areas under the curves (**C**,**D**) derived from an oral glucose tolerance test performed in 6-month-old male and female rats born to saline or DHA supplemented dams. * *p* < 0.05; ** *p* < 0.01; *** *p* < 0.001; (Student’s *t*-test).

While male animals from both DHA-supplemented and non-supplemented dams showed glucose intolerance (Figure 7A,C), only obese female rats from saline-supplemented dams displayed impaired glucose tolerance (Figure 7B,D). In other words, maternal DHA supplementation protected female—but not male—offspring against high-calorie diet-induced glucose intolerance.

#### 3.3.2. Hepatic Liver Accumulation

**Figure 8:**
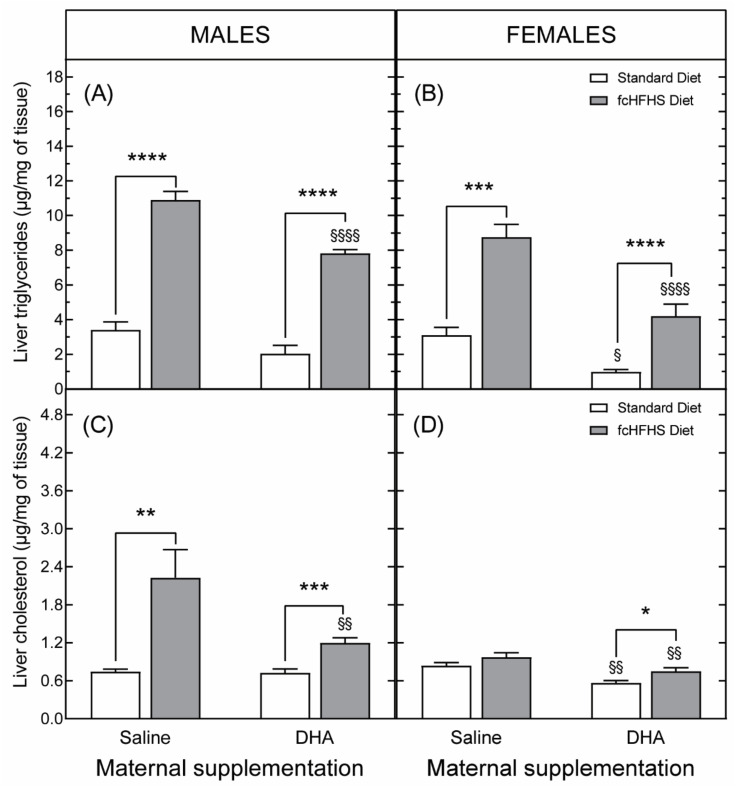
Long-term protective effects of DHA maternal supplementation during gestation and lactation against hepatic steatosis in the offspring. Data correspond to the concentrations of triglycerides (**A**,**B**) and cholesterol (**C**,**D**), determined in livers from 6-month-old male and female rats born to saline- or DHA-supplemented dams. * *p*< 0.05; ** *p* < 0.01; *** *p* < 0.001; **** *p* < 0.0001 (Student’s *t*-test); ^§^ *p* < 0.05; ^§§^ *p* < 0.01; ^§§§§^ *p* < 0.0001 compared to rats born to saline-supplemented dams exposed to the same diet (standard chow or fcHFHS diet) after weaning (two-way ANOVA).

Maternal DHA supplementation also protected both male and female 6-month-old animals from the accumulation of hepatic triglycerides induced by the consumption of the fcHFHS diet (Figure 8A,B). Nevertheless, sex differences were observed in liver cholesterol, both in response to maternal DHA supplementation and in response to the fcHFHS diet. Namely, obese male animals showed an increase in hepatic cholesterol levels that was partially ameliorated by maternal DHA supplementation (Figure 8C). In contrast, control and obese female rats born to saline-supplemented dams exhibited the same accumulation of cholesterol (Figure 8D). Surprisingly, obese female rats from DHA-supplemented dams showed a greater concentration of hepatic cholesterol than their non-obese, DHA-supplemented counterparts. However, these levels were still lower than those of female rats from the SAL-STD and SAL-fcHFHS diet groups (Figure 8D).

#### 3.3.3. Hepatic Gene Expression

**Figure 9:**
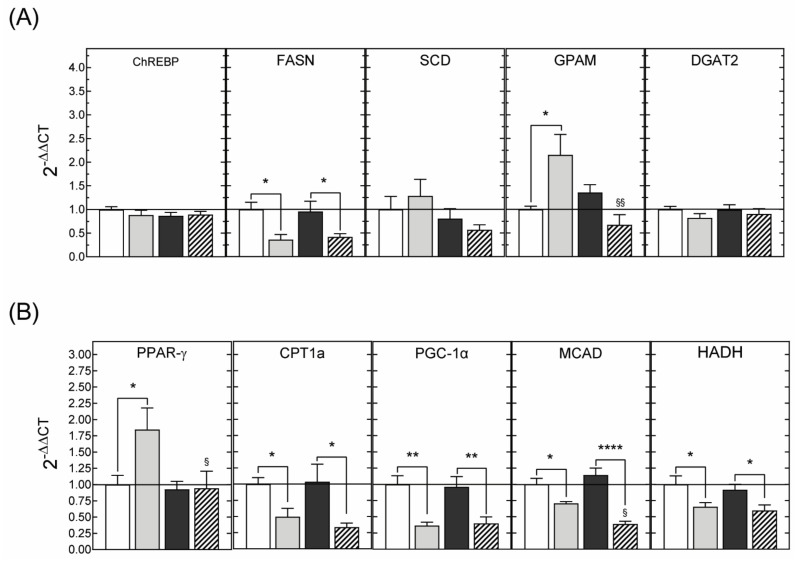
Expression levels of mRNAs encoding proteins involved in de novo lipogenesis (**A**) or fatty acid oxidation (**B**) in liver samples from 6-month-old male rats born to dams supplemented with saline or with DHA during gestation and lactation. The offspring were fed standard chow or rendered obese by exposure to a fcHFHS diet from weaning. Variations in gene expression were calculated by the 2^−ΔΔCT^ method using the expression in animals of the SAL-STD group (white bars) as a calibrator. Grey bars, SAL-fcHFHS group; black bars, DHA-STD group; stripped bars, DHA-fcHFHS diet group. * *p* < 0.05; ** *p* < 0.01; **** *p* < 0.0001 (Student’s *t*-test); ^§^ *p* < 0.05 ^§§^ *p* < 0.01 compared to animals born to saline supplemented dams that were exposed to the same diet (standard or fcHFHS diet), after weaning (two-way ANOVA with maternal supplementation and postnatal diet as factors).

**Figure 10:**
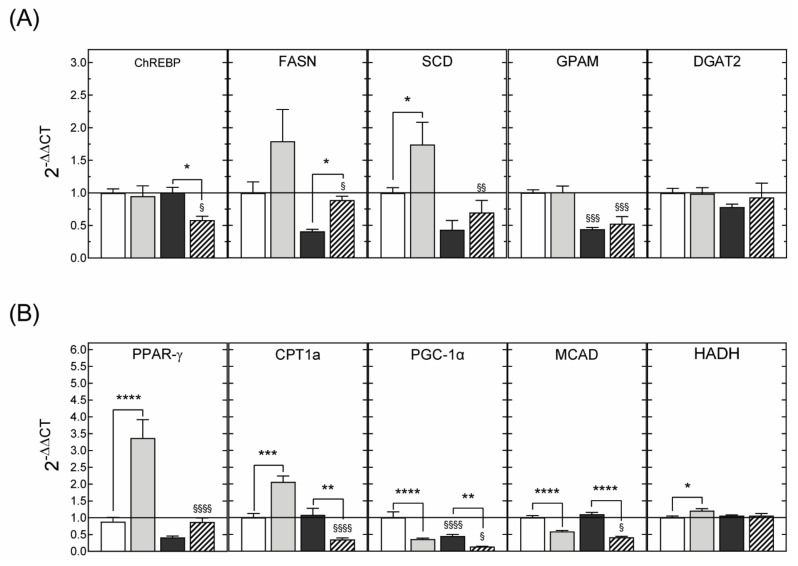
Expression levels of mRNAs encoding proteins involved in de novo lipogenesis (**A**) or fatty acid oxidation (**B**) in liver samples from 6-month-old female rats born to dams supplemented with saline or with DHA during gestation and lactation. The offspring were fed standard chow or rendered obese by exposure to a fcHFHS diet from weaning. Variations in gene expression were calculated by the 2^−ΔΔCT^ method using the expression in animals of the SAL-STD group (white bars) as a calibrator. Grey bars, SAL-fcHFHS group; black bars, DHA-STD group; stripped bars, DHA-fcHFHS diet group. * *p* < 0.05; ** *p* < 0.01; *** *p* < 0.001; **** *p* < 0.0001 (Student’s *t*-test); ^§^ *p*< 0.05 ^§§^ *p* < 0.01; ^§§§^ *p* < 0.001; ^§§§§^ *p* < 0.0001 compared to animals born to saline supplemented dams that were exposed to the same diet (standard or fcHFHS diet), after weaning (two-way ANOVA with maternal supplementation and postnatal diet as factors).

Finally, in agreement with the results obtained in 3-month-old animals, there was a reduction in the hepatic expression levels of genes regulating beta oxidation in both males (Figure 9) and females (Figure 10).

## 4. Discussion

The prevalence of obesity is increasing throughout the world, making this disorder a major public health concern. Although obesity is eventually the result of an imbalance between energy consumption and energy expenditure, numerous epidemiological and experimental studies have shown that the nutritional environment during gestation and during the very first stages of postnatal life plays a key role in determining susceptibility to obesity, as well as to developing metabolic pathologies in adulthood. The aim of this study was to investigate to what extent maternal DHA supplementation during gestation and lactation in the rat protects the offspring against metabolic disorders induced by a high-calorie diet. We focused on DHA because supplementation with this polyunsaturated fatty acid is a common clinical practice used for pregnant women [10,12,13]. Furthermore, it has been shown that the consumption of polyunsaturated fatty acids (PUFAs) reduces body weight [17,18], decreases blood triglyceride levels [36,37], improves carbohydrate homeostasis [38,39], and limits increases in body fat and the development of hepatic steatosis when consuming a high fat diet [20,21]. Based on these observations, we hypothesized that maternal DHA supplementation could positively program the metabolism of the offspring, allowing them to better cope with an obesogenic diet.

Evaluation of the anthropometric characteristics and blood metabolic profile of animals exposed to the fcHFHSD diet from weaning showed that maternal DHA supplementation did not reduce weight gain, fat mass accumulation or the increase in circulating triglyceride levels induced by consumption of a hypercaloric diet. In contrast, obese rats from DHA-supplemented dams showed lower levels of triglycerides and cholesterol in the liver and better glucose tolerance than their saline-supplemented counterparts. These results show that maternal DHA supplementation induces physiological changes in the offspring that are protective against the development of hepatic steatosis and glucose intolerance induced by high-calorie feeding.

Our observations on the lack of effect of maternal DHA supplementation on body weight, body fat, and blood triglycerides in the offspring were consistent with the results of previous studies in humans [40,41,42] and rats [43] showing that the intake of omega-3 PUFAs during the perinatal period had no influence on these parameters. It should be noted, however, that some human studies have documented an inverse relationship between maternal circulating levels of n-3 PUFAs and both body and abdominal fat mass in the offspring [44,45]. These divergent results may be explained in part by differences in the methods of n-3 long chain (LC)-PUFAs supplementation, variability in the techniques used to document body measurements, and the age at which these measurements were made. Additionally, the existence of genetic polymorphisms in the fatty acid desaturase (FADS) gene that catalyze the biosynthesis of unsaturated fatty acids (HUFAs) from PUFAs could be a factor [46].

Animals of the SAL-fcHFHS diet group, had a clear obese phenotype and high serum insulin levels but displayed identical blood glucose concentrations to those of the SAL-STD group both under ad libitum feeding conditions and after fasting. A lack of correlation between circulating glucose levels and the increase in body fat induced by the consumption of a high-fat or high-sugar diet has been reported by other authors [47,48]. It has been suggested that this dissociation could be explained by differences in the composition of the diet or by the level of propagation of insulin resistance, i.e., only in the liver or in the liver and at the peripheral level (skeletal muscle). Alternatively, in our experiments, the obesity-associated hyperglycemia could be masked by circadian variations in blood glucose levels that are at their peak during the early hours of the dark cycle [49], i.e., the time at which the animals were sacrificed. Additional studies are needed to determine which of these two mechanisms underpin our results.

The protective effects of maternal DHA supplementation against glucose intolerance in obese animals was accompanied by a decrease in serum insulin levels, suggesting that maternal DHA supplementation might protect the offspring from developing glucose intolerance by increasing insulin sensitivity. It is also worth noting that non-obese animals from DHA-supplemented dams displayed elevated insulin levels compared to non-obese animals from saline-supplemented dams. The enhanced release of insulin under acute exposure to DHA is well documented [50,51], and is generally accepted as the principal mechanism of the beneficial effects of DHA on insulin resistance. Our results therefore indicated that the protective effects of maternal DHA supplementation against the fcHFHS diet-induced glucose intolerance might also be underpinned by a DHA insulinotropic action.

Feeding a high-calorie diet leads to the development of hepatic steatosis. The accumulation of lipids, mainly in the form of triglycerides, which characterizes this pathological condition, is the consequence of the alteration of one or more liver metabolic processes including excessive lipid entry from the diet, increased de novo fatty acid synthesis, impaired lipid export into the bloodstream, and reduced metabolism through β-oxidation [52,53]. It is generally agreed that the protective effects of the consumption of DHA against the development of hepatic steatosis result mainly from the inhibition of lipogenesis combined with an increase in β-oxidation [25,54,55]. To determine whether the reduction of hepatic steatosis in animals of the DHA-fcHFHS diet group could be explained by the same mechanisms, we assessed the hepatic expression of several genes involved in the regulation of these metabolic pathways using real time PCR. The results of these experiments showed that maternal DHA supplementation blocked the fcHFHS diet-induced increase in the expression of lipogenesis-promoting genes and reduced the levels of mRNAs-encoding genes that positively regulate triglyceride synthesis in both obese and non-obese 3-month-old animals. Therefore, the same mechanisms, operating against hepatic steatosis after the dietary consumption of DHA, seem to be activated in adult offspring rats born to DHA-supplemented dams, despite the fact that they displayed identical circulating DHA levels to animals from non-supplemented dams.

However, in contrast to reports in the literature indicating that omega-3 fatty acids promote lipid oxidation [25,55], the RT-PCR analyses that we carried out showed that maternal DHA supplementation resulted in reduced hepatic expression of genes involved in fatty acid oxidation. Several explanations can be put forward to account for these paradoxical observations. First, conflicting results exist about the impact of hepatic steatosis on the expression of genes regulating β-oxidation. For example, a decrease in CPT1a expression, coupled with enhanced long-chain acyl-CoA dehydrogenase (LCAD) and HADH mRNAs levels, was detected in liver biopsies from patients with non-alcoholic fatty liver disease [56]. Inhibition of CPT1a expression is indicative of decreased β-oxidation, whereas the increased expression of LCAD and HADH suggested that lipid oxidation in these patients was enhanced. These contradictory results were consistent with other studies showing enhanced [57,58], decreased [59], or unchanged [60] fatty acid oxidation in the liver of patients with hepatic steatosis. Second, whether increased lipid oxidation in hepatic steatosis has beneficial effects at all has been challenged [61,62]. In fact, fatty liver has been associated with impaired mitochondrial activity, such that enhanced β-oxidation can lead to excessive production of reactive oxygen species (ROS) and the activation of fatty acid oxidation in cytochromes and peroxisomes and, therefore, to hepatic damage by oxidative stress and inflammation. Third, a comparative study of the β-oxidation capacity of DHA and EPA in the liver showed that EPA is much more effective in increasing mitochondrial fatty acid oxidation than DHA [63]. Since the fish oil-extract we used contains 7 times less EPA than DHA, our results could be explained by the low proportion of EPA in relation to DHA. Fourth, fatty acids derived from triglyceride lipolysis are converted to acetyl-CoA whose transformation by carboxylation to malonyl-CoA is the committed step in the de novo fatty acid synthesis. Yet, rats from DHA-supplemented dams also exhibited a reduced expression of the GPAM and DGAT2 genes whose enzymatic products catalyze triglyceride synthesis. Therefore, low levels of expression of genes regulating fatty acid oxidation could be a consequence of a reduction in the amount of substrate required to initiate β-oxidation. Finally, our results are consistent with other studies showing that EPA and DHA reduced HFD-induced hepatic steatosis without affecting the expression of genes involved in β-oxidation [64].

The deleterious effects of obesity increase with age [65,66] and differ by gender [67,68,69]. With the aim of determining the impact of these parameters on the protective effect of maternal DHA supplementation, in a second series of experiments, the metabolic characteristics of six-month-old male and female animals born to DHA-supplemented dams and subjected to the fcHFHS diet were analyzed. The results of these studies showed that the protective effects of maternal DHA supplementation against hepatic steatosis were common to both sexes. However, only female rats from DHA-supplemented dams were protected against the development of glucose intolerance associated with the exposure to the fcHFHS diet. This latter result could be interpreted as being evidence of sexual dimorphism in the effects of DHA, as reported in previous studies [70,71]. However, females are more insulin sensitive and glucose tolerant than males [72,73]. Thus, the lack of glucose intolerance in female rats in the DHA-fcHFHS diet group may also be linked to intrinsic metabolic differences between males and females. In support of this interpretation, exposure to the fcHFHS diet did not result in increased hepatic cholesterol levels in female rats from saline-supplemented dams, whereas in male animals exposed to the same nutritional manipulations, hepatic cholesterol concentrations were significantly increased. Regarding the impact of aging, the results obtained in male animals indicated that the protective effects of maternal DHA supplementation against hepatic steatosis persisted with age, despite the greater accumulation of triglycerides and cholesterol in the liver in 6-month-old obese animals compared to 3-month-old rats of the same experimental group.

Notwithstanding the numerous human studies involving maternal DHA supplementation during pregnancy, very few animal studies have sought to determine the biochemical and molecular mechanisms underlying the putative beneficial effects of this nutritional intervention—particularly with regard to the metabolism of the offspring. To our knowledge, there have only been two studies in which the metabolic phenotype of offspring exposed in utero to DHA was investigated. In these studies, carried out in hamsters, it was shown that maternal consumption of an EPA/DHA-enriched diet during gestation and lactation protected the offspring against increases in plasma glucose levels, hepatic triglyceride secretion and lipemia induced by feeding a high-fat diet via the enhancement of tricarboxylic acid cycle efficiency [74,75]. However, in these studies, DHA was provided via the diet, in contrast to most clinical nutritional interventions, in which DHA is provided orally in well-defined amounts. The results presented herein thus extend and deepen the limited knowledge on the consequences of maternal DHA supplementation on the metabolism of the offspring by showing, on the one hand, the protective effects of this nutritional intervention against the development of glucose intolerance and hepatic steatosis associated with obesity and, on the other hand, the existence of sex-related differences in the effects of early life exposure to DHA.

It is remarkable that the inhibition of lipogenesis underlying the protective effects of DHA against fatty liver in obese individuals—as described in intervention studies in human and rodents [25,76,77]—also acts to prevent the development of high-calorie diet-induced hepatic steatosis when DHA supplementation is provided during development via the mother as shown in the present study. This lends our model and results a translational research value and raises the question of the mechanisms by which temporary exposure to DHA during embryogenesis and neonatal life downregulates the expression of lipogenic genes in the long term. The establishment of a particular epigenetic profile following the in utero/neonatal exposure to DHA could be one of these mechanisms. Actually, maternal DHA supplementation during the last trimester of pregnancy has been shown to increase methylation levels at the differentially methylated region (DMR) of insulin growth factor 2 (IGF2) in the offspring’s cord blood mononuclear cells [78]. Likewise, lower methylation levels of differentially methylated regions located in genes involved in the regulation of lipid exchange, plasma membrane function, food intake, immune function, and neurodevelopment were detected in blood spots collected at birth from babies born to mothers supplemented with DHA from 20 weeks of gestation to delivery [79]. Interestingly, some of these changes were sex-dependent and persisted until 5 years of age. However, other studies failed to detect differences in DNA methylation levels in CD4 ± T-cells isolated from the cord blood of babies born to mothers that received DHA from 20 weeks of gestation until delivery [80]. In rodents, maternal DHA supplementation has been shown to restore intrauterine growth restriction-induced decreased levels of histone H4K20 methylation at the PPARγ promoter gene in the lung to normal values [81]. As an initial approach to determine if the protective effects of maternal DHA supplementation against the development of hepatic steatosis could involve a modification of the epigenome, we used western blot to examine two post-translational histone modifications related to transcriptional repression (H3K9me3 and H3K27me3) and two histone modifications related to transcriptional activation (H3K4me3 and H3K14me3). No differences in these epigenetic marks were detected between the different groups (unpublished observations). However, these negative results did not exclude the involvement of other epigenetic mechanisms (DNA methylation, miRNAs expression) in the protective effects of DHA.

## 5. Conclusions

In summary, the present study provided compiling evidence to show that maternal DHA supplementation in rats during pregnancy protected the offspring against glucose intolerance and hepatic steatosis induced by the consumption of a hypercaloric diet. The beneficial effects on glucose homeostasis declined with age in a sex-dependent manner. However, the preventive action against hepatic steatosis persisted at least until 6 months of age and was associated with decreased hepatic expression of lipogenic genes. These results indicate that maternal DHA supplementation during pregnancy can program a healthy metabolic state in the offspring which protects against the deleterious effects of an obesogenic diet. The involvement of epigenetic mechanisms in the beneficial programming action of DHA deserves further investigation.

## Figures and Tables

**Table 1:** Sequences of primers used for real time RT-PCR analyses.

Gene Name	Symbol	Forward Primer	Reverse Primer
Carbohydrate response element binding protein	ChREBP	GTACTGTTCCCTGCCTGCTCTC	CCCTCTGTGACTGCCCTTGTG
Fatty acid synthase	FASN	CGCCGTGGTGCTGGAGATTG	CTTGCCGAGGTTGGTGAGGAAG
Stearoyl-coenzyme A desaturase	SCD	GGAGCCACAGGACTTACAAGG	CGCACAAGCAGCCAACCC
Glycerol-3-phosphate acyltransferase, mitochondrial	GPAM	TGGAGTGTGGCAAGAGGCGTTATC	TTCGGCAGCAGCAGCATCAGG
Diacylglycerol O-acyltransferase 2	DGAT2	CCTCATCGCTGCCTACTCC	TGAGCCAGGTGACAGAGAAG
Peroxisome proliferator-activated receptor delta	PPARγ	GGAATTAGATGACAGTGACTTGGC	GGAGCACCTTGGCGAACAG
Carnitine palmitoyltransferase 1A	CPT1a	TGCCTGCCAGTTCCATTAAGC	GTCTCACTCCTCTTGCCAACAG
Peroxisome proliferator-activated receptor gamma, co-activator 1alpha	PGC-1α	ACACCGCACACATCGCAATTC	TTCGTCCCTCTTGAGCCTTTCG
Medium chain acyl coenzyme A dehydrogenase	MCAD	GAGGCTAGAAGGTCCTGAGAAGTG	TCTGCTGCTCCGTCAACTGG
Hydroxyacyl-Coenzyme A dehydrogenase	HADH	CTCCATGTCCTCCTCTTCCTCTGC	CAGCCCGCCGCCGATGAC
18S ribosomal RNA	18S	GATGCGGCGGCGTTATTCC	CTCCTGGTGGTGCCCTTCC
Beta-2 microglobulin	β2M	GATGGCTCGCTCGGTGAC	CGTAGCAGTTGAGGAAGTTGG

**Table 2:** Anthropometric and metabolic characteristics of 3-month-old male rats born to saline or DHA-supplemented dams.

	Supplemented Saline		Supplemented DHA	
	Standard Diet	FcHFHS Diet	Standard Diet	FcHFHS Diet
Body weight (g)	510 ± 12	600 ± 16 ****	477 ± 10	599 ± 15 ****
Adipose Index	4.40 ± 0.20	7.36 ± 0.31 ****	4.76 ± 0.23	6.66 ± 0.32 ***
Leptin (ng/mL)	12.43 ± 0.83	33.10 ± 4.31 ****	13.72 ± 2.29	34.58 ± 5.44 **
Triglycerides (mg/dL)	95.48 ± 10.45	157.49 ± 15.70 **	91.27 ± 5.11	171.58 ± 28.29 *
Cholesterol (mg/dL)	67.54 ± 3.54	77.24 ± 2.96	64.72 ± 4.64	72.38 ± 4.32
Glucose (mg/dL)	189.54 ± 5.43	206.66 ± 6.27	197.20 ± 8.26	204.16 ± 9.50

* *p* < 0.05, ** *p* < 0.01, *** *p* < 0.001, **** *p* < 0.0001 compared to animals fed standard diet of the same experimental group (saline or DHA supplemented). All statistical comparisons were done by two-way ANOVA using maternal supplementation and postnatal diet as factors followed by Šídák’s multiple comparisons test.

**Table 3:** Anthropometric and metabolic characteristics of 6-month-old male and female rats born to saline- or DHA-supplemented dams.

MALES				
	Supplemented Saline		Supplemented DHA	
	Standard Diet	fcHFHS Diet	Standard Diet	FcHFHS Diet
Body weight (g)	633 ± 19	754 ± 24 ***	630 ± 20	770 ± 24 ***
Adipose Index	6.51 ± 0.43	9.79 ± 0.37 ****	7.05 ± 0.11	10.72 ± 0.85 ***
Leptin (ng/mL)	14.99 ± 1.76	26.04 ± 1.91 **	15.69 ± 1.37	30.27 ± 3.55 ***
Triglycerides (mg/dL)	101.44 ± 10.96	151.09± 20.08 *	65.61 ± 7.49	119.45± 18.16 *
Cholesterol (mg/dL)	73.62 ± 5.54	74.83 ± 2.90	79.80 ± 6.11	72.94 ± 2.85
Glucose (mg/dL)	155.24 ± 9.04	177.42 ± 22.61	175.40 ± 27.71	209.04 ± 14.87
Insulin (ng/mL)	1.43 ± 0.18	2.99 ± 0.67 *	1.26 ± 0.22	4.08 ± 0.92 **
**FEMALES**				
	Supplemented Saline		Supplemented DHA	
	Standard Diet	fcHFHS Diet	Standard Diet	FcHFHS Diet
Body weight (g)	349 ± 80	451 ± 19 ****	357 ± 11	407 ± 26 ****
Adipose Index	7.33 ± 0.56	22.70 ± 0.42 ****	7.67 ± 0.42	10.00 ± 1.59 ***
Leptin (ng/mL)	6.53 ± 0.74	22.70 ± 3.04 ***	6.81 ± 1.05	16.13 ± 3.10 *
Triglycerides (mg/dL)	125.17 ± 13.27	339.99 ± 98.61 *	140.59 ± 20.99	285.48 ± 36.99 *
Cholesterol (mg/dL)	61.13 ± 3.91	85.11 ± 5.24	84.13 ± 4.10 ^§§^	96.36 ± 3.83
Glucose (mg/dL)	171.65 ± 4.17	182.70 ± 10.07	183.89 ± 11.55	182.30 ± 13.42
Insulin (ng/mL)	0.93 ± 0.18	3.40 ± 0.76 **	1.45 ± 0.25	2.50 ± 0.57

* *p* < 0.05, ** *p* < 0.01, *** *p* < 0.001, **** *p* < 0.0001 compared to animals fed standard diet of the same sex and the same experimental group (supplemented saline or DHA, Student’s *t*-test). ^§§^ *p* < 0.01 compared to the supplemented saline standard diet group (two-way ANOVA using maternal supplementation and postnatal diet as factors followed by Šídák’s multiple comparisons test). Data correspond to six animals per group.

## Data Availability

Data are available from the corresponding author on reasonable request.
